# Targeting TGF-β signaling in the multiple myeloma microenvironment: Steering CARs and T cells in the right direction

**DOI:** 10.3389/fcell.2022.1059715

**Published:** 2022-12-12

**Authors:** Priyanka S. Rana, David C. Soler, Jeries Kort, James J. Driscoll

**Affiliations:** ^1^ Division of Hematology and Oncology, Department of Medicine, Case Western Reserve University, Cleveland, OH, United States; ^2^ Case Comprehensive Cancer Center, Cleveland, OH, United States; ^3^ The Brain Tumor and Neuro-Oncology Center, The Center of Excellence for Translational Neuro-Oncology, Department of Neurosurgery, Case Western Reserve University, Cleveland, OH, United States; ^4^ Adult Hematologic Malignancies and Stem Cell Transplant Section, Seidman Cancer Center, University Hospitals Cleveland Medical Center, Cleveland, OH, United States

**Keywords:** transforming growth factor-β, multiple myeloma, T-cell, drug resistance, CAR (chimeric antigen receptor) T-cells, bispecific T-cell engager (BiTE) therapy

## Abstract

Multiple myeloma (MM) remains a lethal hematologic cancer characterized by the expansion of transformed plasma cells within the permissive bone marrow (BM) milieu. The emergence of relapsed and/or refractory MM (RRMM) is provoked through clonal evolution of malignant plasma cells that harbor genomic, metabolic and proteomic perturbations. For most patients, relapsed disease remains a major cause of overall mortality. Transforming growth factors (TGFs) have pleiotropic effects that regulate myelomagenesis as well as the emergence of drug resistance. Moreover, TGF-β modulates numerous cell types present with the tumor microenvironment, including many immune cell types. While numerous agents have been FDA-approved over the past 2 decades and significantly expanded the treatment options available for MM patients, the molecular mechanisms responsible for drug resistance remain elusive. Multiple myeloma is uniformly preceded by a premalignant state, monoclonal gammopathy of unknown significance, and both conditions are associated with progressive deregulation in host immunity characterized by reduced T cell, natural killer (NK) cell and antigen-presenting dendritic cell (DC) activity. TGF-β promotes myelomagenesis as well as intrinsic drug resistance by repressing anti-myeloma immunity to promote tolerance, drug resistance and disease progression. Hence, repression of TGF-β signaling is a prerequisite to enhance the efficacy of current and future immunotherapeutics. Novel strategies that incorporate T cells that have been modified to express chimeric antigen receptor (CARs), T cell receptors (TCRs) and bispecific T cell engagers (BiTEs) offer promise to block TGF-β signaling, overcome chemoresistance and enhance anti-myeloma immunity. Here, we describe the effects of TGF-β signaling on immune cell effectors in the bone marrow and emerging strategies to overcome TGF-β-mediated myeloma growth, drug resistance and survival.

## 1 Biologic effects of the TGF-β signaling pathway

The transforming growth factor-β (TGF-β) pathway governs cellular growth, differentiation, apoptosis, invasion, and immunity (Massagué, 2008; Moses et al., 2016). A better understanding of the mechanisms that regulate the TGF-β pathway and downstream signaling steps has produced a coherent roadmap of the signaling cascade, as well as crosstalk with other pathways (Moustakas and Heldin, 2009; Massagué, 2012; Batlle and Massagué, 2019). Obstacles still exist in determining the precise role of TGF-β that originates from the context-dependent nature on various cell types. TGF-β1, 2 and 3 are evolutionarily conserved, secreted, isoforms that control cellular behavior under physiologic and pathologic conditions (Heldin and Moustakas, 2016; Malek et al., 2019; Tzavlaki and Moustakas, 2020; Kim et al., 2021). Activated TGF-β binds type I and type II TGF-β receptor kinase receptors (TGF-βRI and TGF-βRII), respectively. TGF-β binding promotes TGF-βRII heterotetramerization and phosphorylation to activate TGF-βRI, followed by Smad2/3 phosphorylation with subsequent activation of downstream signaling pathways. TGF-β has been shown to elicit both tumor suppressive and tumor promoting activities (Chen W. J et al., 2003; Fantini et al., 2004; Heldin and Moustakas, 2016; Dahmani and Delisle, 2018). In healthy cells, TGF-β functions as a potent growth inhibitor while in developed tumors, which produce excess TGF-β, it functions as a growth promoter. However, the homeostatic action of TGF-β is thwarted in oncogenically-activated cells. Within the tumor microenvironment (TME), TGF-β promotes cancer progression and metastasis (Swamydas et al., 2022). At later stages of disease, TGF-β targets vascular, fibroblastic, and immune cells (Blobe et al., 2000; Bhowmick et al., 2004; Massagué, 2012). SMAD is a canonical TGF-β signaling effector that controls metastasis and immune regulation (Chen et al., 2005; Laouar et al., 2005; Yu et al., 2006; Takimoto et al., 2010). TGF-β also modulates the epithelial to mesenchymal transition (EMT) in which epithelial cells convert to a mesenchymal phenotype that increases tumor growth (Blobe et al., 2000; Kim et al., 2020). Although the expression of TGF-β and TGF-βR1/II is widespread, TGF-β activation is localized to sites where the cytokine is released from latency (Shi et al., 2011). TGF-β is stored as a latent complex with the prodomain intact within the ECM. TGF-β1 activation requires the binding of α_v_ integrin to an RGD sequence in the prodomain while TGF-β resides within the ECM with latent TGF-β (LTGFB) binding proteins. LTGFB is activated by several signals, e.g., integrins, thrombospondin and proteases. The ECM1 protein inhibits LTGFB activation and depletion of ECM1 leads to a massive R-Smads activation in the liver (Li et al., 2021). In healthy tissues, TGF-β maintains homeostasis to prevent tumor formation (Bhowmick et al., 2004; Mukherjee et al., 2010; Macfarlane et al., 2017).

## 2 Role of TGF−β signaling in cancer

**FIGURE 1:**
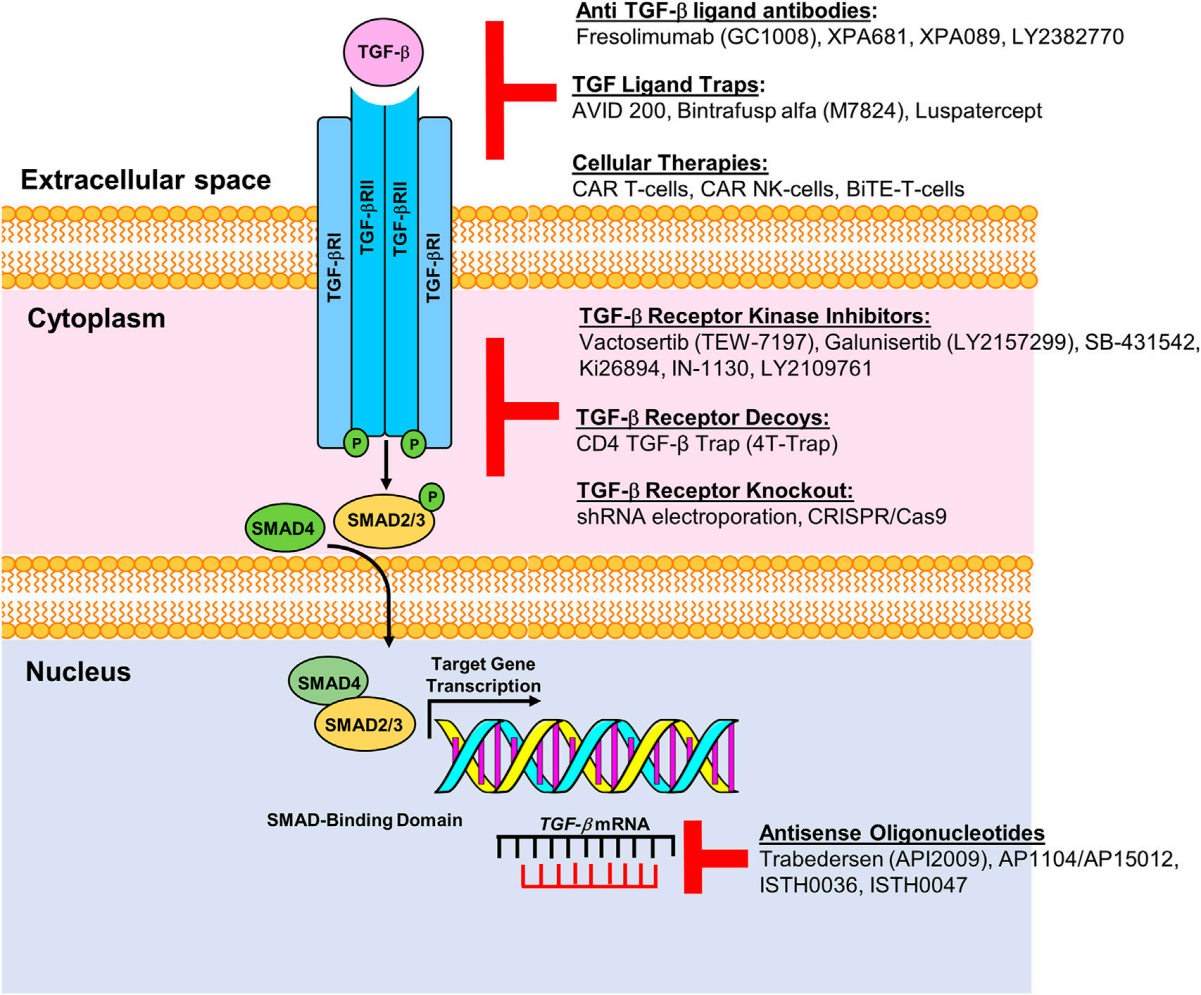
Pharmacologic and immunologic blockade of the TGF-β pathway. Schematic representation of multiple modalities developed or in development to target components of the TGF-β signaling pathway. Current roadblocks due to TGF-β mediated immune suppression can be overcome by targeting the TGF-β receptor with ligand antibodies, ligand traps or by inhibiting TGF-β receptors using specific kinase inhibitors, receptor decoys or by knocking out the TGF receptor gene. Antisense oligonucleotides, e.g., AP11014 and AP15012, can silence or suppress TGF-β at the transcriptional level. Figure is adapted from Kim et al., 2021.

Cancer cells develop resistance to the suppressive effects of TGF-β through mutation or epigenetic modification (Ikushima and Miyazono, 2010). For example, TGF-β1 signaling is hyperactivated in breast cancer cells and promotes tumorigenesis (Tang et al., 2018). SMAD7 is an antagonist of TGF-β1 signaling and complexes with the TGF-βRI to inhibit downstream signaling and pro-tumorigenic transcriptional activation. Proteins that regulate SMAD7 activity act to attenuate TGF-β1-mediated aggressive phenotypes in breast cancer (Hu et al., 2021). In colon cancer, *SMAD7* deletion predisposes to a favorable prognosis, while *SMAD7* amplification is linked to poor outcomes (Boulay et al., 2003). While TGF-β signaling activates Smad2/3, BMP (bone morphogenetic protein) signaling cascade activates Smad1/5/8. Both cascades share Smad4 but are inhibited by Smad6/7, while at the transcriptional level, TGF-β Smad4 complexes with Smad2/3, and BMP Smad4 complexes with Smad 1/5/8 (Dituri et al., 2019). BMP-7 deficiency accelerates tumorigenesis and negatively correlates with patient outcome (Tang et al., 2018). Prostate carcinoma demonstrates reduced *BMP7* transcript levels relative to normal prostate gland. Recombinant BMP7 reduced tumor burden and metastasis in mice. Since the TGF-β signaling pathway represents an attractive target for cancer therapy, numerous modalities have been designed or are in development as anti-cancer strategies (Figure 1).

## 3 Tumor intrinsic effects of TGF-β confers drug resistance in multiple myeloma

TGF-β1 not only contributes to malignant progression but also promotes the development of drug resistance (Coşkun et al., 2006; Dong and Blobe, 2006). Elevated levels of TGF-β1 in MM patient serum correlates with the emergence of drug resistance, tumor progression and a poor prognosis (Coşkun et al., 2006). The proteasome is an evolutionarily conserved cylindrical structure of high molecular weight that demonstrates multiple endopeptidase activities (Driscoll, 1994; Driscoll et al., 2012). Plasma cells are protein-synthesizing factories that secrete copious amounts of antibodies and therefore rely heavily on protein homeostatic mechanisms. The ubiquitin-proteasome system degrades the vast majority of proteins in eukaryotic cells. Malignant plasma cells are exquisitely sensitive to agents that disrupt proteostasis. Proteasome inhibitors have been FDA-approved and are now frontline treatment for newly diagnosed and relapsed and/or refractory multiple myeloma (RRMM) as well as for Mantle Cell Lymphoma (Driscoll and Woodle, 2012). Moreover, the proteasome inhibitors bortezomib and carfilzomib have shown significant promise in targeting plasma cells that secrete HLA-antibodies and hinder organ transplantation (Tremblay et al., 2020; Woodle et al., 2020). TGF-β/Smad signaling has been shown to regulate expression of the proteasome catalytic subunit *PSMB5* (Lai et al., 2018) and a number of studies suggest that *PSMB5* contributes to proteasome inhibitor resistance (Oerlemans et al., 2008; Ignatz-Hoover et al., 2022). Studies in bortezomib-resistant myelomonocytic cells revealed *PSMB5* overexpression, mutations within the highly conserved bortezomib binding pocket in PSMB5 and that *PSMB5* knockdown restored drug sensitivity. RPMI 8226 cells grown in escalating doses of bortezomib generated drug resistant cells that overexpressed the proteasome β5 catalytic subunit *PSMB5* at the messenger RNA (mRNA) and protein levels (Balsas et al., 2012). Bortezomib resistant lymphoma and leukemia cell lines also exhibit *PSMB5* overexpression (Lü et al., 2008). Increased *PSMB5* expression has been associated with a less favorable outcome in triple negative breast cancer (TNBC), while silencing *PSMB5* sensitized TNBC cells to chemotherapy (Wei et al., 2018).

## 4 TGF-β effect on immune escape in the myeloma microenvironment

**FIGURE 2:**
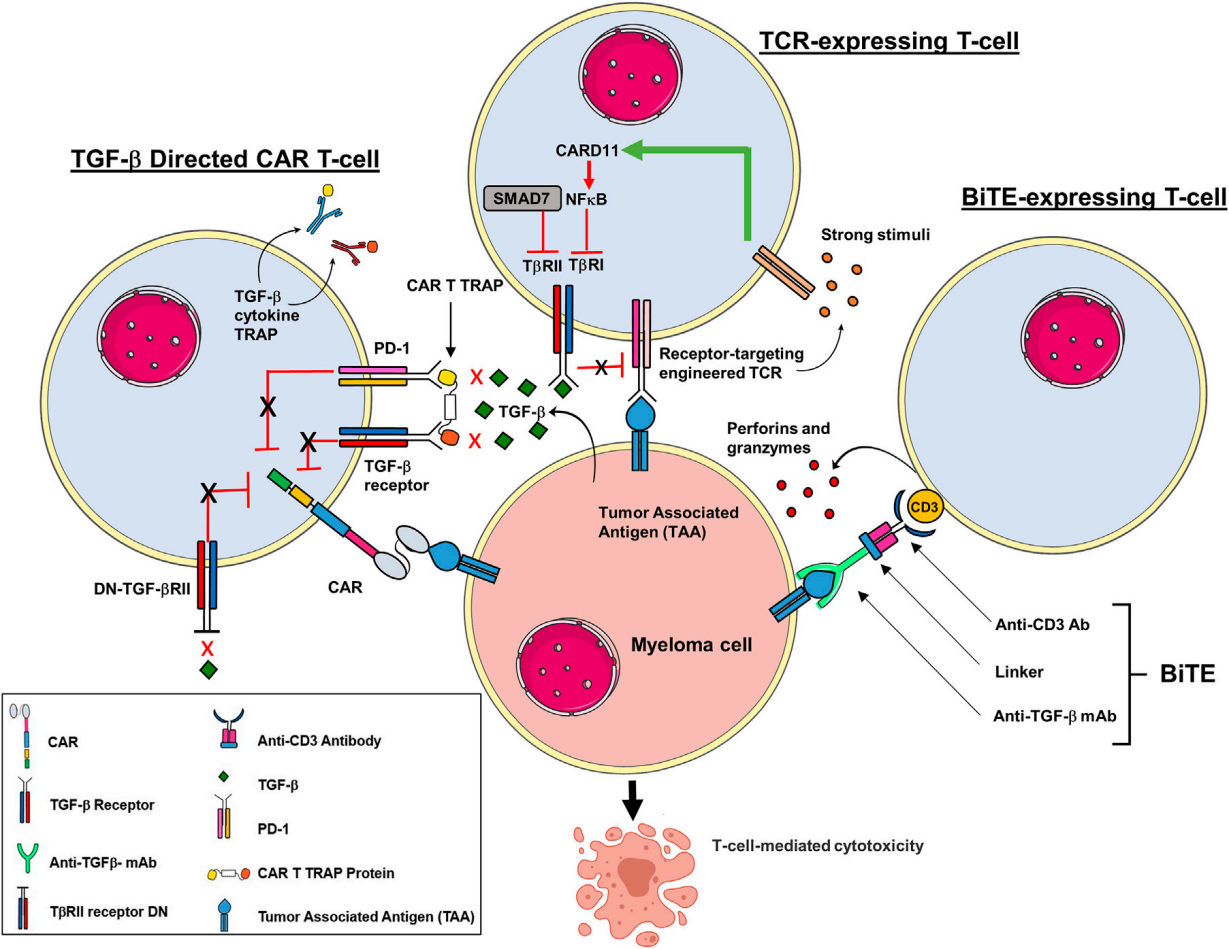
Adoptive T cell strategies to prevent immune suppression by TGF-β in TME. Multiple strategies are in development to modify T cells to target components of the TGB-β signaling pathway. T cells can be engineered to work against the immunosuppressive role of TGF-β, activated to induce a more efficient T cell cytotoxicity and prevent exhaustion. CAR T cells have been engineered to block the inhibitory effects of TGF-β by co-expressing a BCMA construct that expresses a dominant negative (DN) TGF-βRII armor. The DN TGF-βII lacks a functional receptor kinase domain which prevents TGF-β suppression despite prolonged exposure. CAR T cells have also been modified to express a decoy or a cytokine receptor that functionally converts inhibitory signals within the TME into pro-inflammatory signals. In addition, CAR T cells have been engineered to secrete a bispecific antibody that co-targets both PD-1 and TGF-βRII to improve anti-tumor immunity. T cells that express a TCR that engages the TGF-βRII to activate caspase recruitment domain-containing protein 11 (CARD11) and nuclear factor κ-B (NF-κB). Moreover, the inhibitory effects of SMAD7 on TGF-βRI promotes T cell mediated cytotoxicity. BiTE-expressing T cells simultaneous binding to CD3 in the TCR complex and the TGF-β to promote lytic enzymes that mediate CTL activity.

TGF-β regulates immune cell activity and suppresses immunosurveillance (Gorelik and Flavell, 2002; Swamydas et al., 2022) (Figure 2). TGF-β is secreted by malignant plasma cells that are characteristic of MM. TGF-β triggers interleukin (IL)-6 and vascular endothelial growth factor (VEGF) secretion in BM stromal cells (BMSCs), to increase paracrine IL-6 and VEGF-related tumor growth (Urashima et al., 1996; Hayashi et al., 2004). Within the BM, TGF-β regulates cytotoxic T cell and regulatory T cell (Tregs) (Wan and Flavell, 2008), natural killer (NK) cells (Regis et al., 2020), and macrophage activity (Zhang et al., 2016). A primary role of TGF-β is the generation of Tregs which suppress anti-myeloma immunity (Hadjiaggelidou and Katodritou, 2021). Antibodies and pharmacologics that block TGF-β signaling promote CD8^+^ T cell and NK-cell driven anti-tumor responses (Gunderson et al., 2020). Other agents elevate neutrophil-attracting chemokines to promote their recruitment. TGF-β silences IL-2 expression and TGF-β signaling blocks CD8^+^ T cells functional activity (Batlle and Massagué, 2019).

TGF-β stimulates Treg differentiation and expansion to promote immune evasion. TGF-β and IL-2 drive immune suppression by inducing *Foxp3* expression in naïve CD4^+^ T cells (Chen C. H et al., 2003; Fantini et al., 2004). *Foxp3* is a master transcription factor that regulates the Treg program and is induced by TGF-β (Takimoto et al., 2010). However, pro-inflammatory cytokines within the TME counteract TGF-β-driven Treg induction and drive CD4^+^ T cell conversion towards an effector phenotype (Wei et al., 2007; Battaglia et al., 2013). TGF-β reduces the production and activity of NK cells by suppressing IFN-γ as well as expression of the transcription factor T-bet (Batlle and Massagué, 2019). TGF-β also reduces expression of the surface receptor proteins *NKG2D* and *NKp30* to impair recognition of stressed and transformed cells (Castriconi et al., 2003). Dendritic cells (DCs) are antigen-presenting cells (APCs) that have been shown to regulate Th1 and Treg-driven responses. TGF-β inhibits antigen presentation by DCs *in vitro* by suppressing MHC class II levels. Tumor cells induce the release of TGF-β by DCs which promotes the conversion of naïve CD4^+^ T cells into Tregs. Macrophages are polarized towards an M2 phenotype with anti-inflammatory and immune suppressive functions (Boutilier and Elsawa, 2021). Tumor-associated macrophages (TAMs) produce TGF-β and macrophage subsets mobilize TGF-β through integrin αv-β8 and MMP1(Kim et al., 2021). TGF-β acts as a chemoattractant that draws monocytes to inflammatory sites and upregulates adhesion molecules that facilitate monocyte attachment to the ECM. The TGF-β-rich TME further contributes to immune suppression by squelching macrophage activity (Ignatz-Hoover and Driscoll, 2022).

TGF-β1-mediated differentiation of myeloid-derived suppressor cells (MDSCs) leads to a potent anti-tumor phenotype suitable for adoptive T cell therapy (ACT) (Jayaraman et al., 2018). TGF-β controls MDSC differentiation and immunoregulatory activity while MDSCs enhance T cell anti-tumor effects. TGF-β increases monocytic MDSC (Mo-MDSC) expansion, the expression of immunosuppressive molecules, and suppression of CD4⁺ T cell proliferation (Lee et al., 2018).

TGF-β1 has been shown to be a potent enhancer of mRNA expression of BM stroma thrombopoietin (TPO), a commitment of lineage specificity. TPO induces TGF-β receptors I and II on megakaryoblasts at the mid-megakaryopoietic stage; at this stage, TGF-β1 was able to arrest the maturation of megakaryocyte colony-forming units (CFU-Meg) (Sakamaki et al., 1999). TGF-β1 released from destroyed platelets or megakaryocytes stimulates TPO synthesis in BMSC which then stimulates CD34^+^ stem cells to commit to the megakaryocyte lineage and to express TGF-β1 receptors that render them susceptible to suppression by TGF-β1 as part of a feedback regulatory loop. Megakaryocytes are an important component of the TME and directly supporting MM growth. Therapeutic interventions that interfere with MM-megakaryocyte interaction may help control disease (Yaccoby et al., 2005).

## 5 TGF-β effects on T cell senescence and exhaustion

A hallmark of senescence is an accumulation of mutations in the machinery that controls cellular replication. TGF-β exercises its effects on cellular aging in a variety of ways owing to its vast array of pleiotropic effects (Oh and Li, 2013). TGF-β interferes with cell cycle regulators, and by suppressing proliferation factors (Zhang et al., 2017). TGF-β accelerates aging in fibroblasts, bronchial epithelial and cancer cells (Debacq-Chainiaux et al., 2005; Miyazono et al., 2018). Reactive oxygen species (ROS) is a hallmark of senescence and TGF-β increases ROS production within mitochondria of several cells, e.g., hepatocytes, lung epithelia (Albright et al., 2003; Yoon et al., 2005). TGF-β also exerts effects on expression of the telomerase reverse transcriptase (TERT) in fibroblasts and breast cancer cells (Hu et al., 2006; Li H. et al., 2006). The effects of TGF-β are also observed in non-protein entities, e.g., non-coding RNAs and microRNAs (Suzuki and Miyazono, 2011; Suzuki, 2018). TGF-β induces inhibitory Smads (I-Smads), Smad6 and Smad7 expression which contributes to negative feedback that contributes to senescence. TGF-β can be secreted in either an autocrine or paracrine manner.

During thymic development, self-reactive T cells undergo a goldilocks process where they are eliminated to prevent auto-reactive effects (Dzhagalov et al., 2013; Li M. O et al., 2006; Marie et al., 2006). There is evidence that TGF-β can dampen auto-reactive T cells that have avoided depletion during development (Ouyang et al., 2010). This phenomenon has implications for cancer biology, since TGF- β promotes the generation of immunosuppressive Tregs (Josefowicz et al., 2012). TGF-β induces Foxp3 in peripheral Tregs with concomitant expression of IL-2 (Chen W. J. et al., 2003; Davidson et al., 2007; Zheng et al., 2007). TGF-β1 is the predominant isoform secreted by immune cells and its primary role is to maintain a self-tolerant and functional T cell repertoire. However, TGF-β signaling has redundant effects and doubts persist on how Smad-dependent transcriptional activation controls T cell programs (Takimoto et al., 2010; Gu et al., 2012).

Importantly, TGF-β impacts T cell activation by inhibiting TCR signaling (Chen, C. H. et al., 2003) and downregulates expression of lineage-defining transcription factors, e.g., T-bet and GATA-3, which are critical for Th1 and Th2 CD4^+^ T-cell differentiation, respectively (Gorelik et al., 2000; Neurath et al., 2002). In contrast to inhibiting certain programs in effector T cells, TGF-β also enhances Th17 lineage T cell differentiation (Veldhoen et al., 2006). Another subset of CD4^+^ T cells affected by TGF-β are the Th9 cells which secret pro-inflammatory IL-9 when T cells are exposed to TGF-β and IL-4 (Jabeen and Kaplan, 2012).

Exhausted T cells display reduced proliferative capacity and impaired function activity, similar to that seen in viral infections (Gallimore et al., 1998; Urbani et al., 2006). Several studies have implicated TGF- β as having a role to induce T cell exhaustion that ultimately leads to T cell death (Tinoco et al., 2009). However, the specifics on this phenomenon have not been yet elucidated. Interestingly, B cell malignancies produce IL-10 and TGF-β (Gupta et al., 2012; Huai et al., 2021), while non-Hodgkin’s lymphoma cells upregulate CD70 expression on T cells through TGF-β (Yang et al., 2014). However, CD70 expression on MM cells is non-uniform (Zheng et al., 2013; Timmers et al., 2019). TGF-β also elevates the inhibitory markers CD39 and CD73 expressed on CD8^+^ T cells, limiting T cell activity (Li et al., 2017). TGF-β secreted from TAMs inhibits INF-γ and granzyme B release. In contrast, anti-TGF-β antibodies or a dominant negative (DN) TGF-β receptor have been shown to increase T cell activity (Wang et al., 2019).

## 6 CAR T cells directed against the TGF-β pathway

CAR T cells have been shown high response rates for RRMM patients (Berdeja et al., 2021; Munshi et al., 2021) (Fig. 2). However, while a promising new anti-cancer strategy, CAR T cells present unique challenges (Maus et al., 2014; Rana et al., 2022). The TME is highly immunosuppressive and is comprised of many different cells that directly or indirectly impede the cytotoxic activity of T cells. TGF-β is secreted by many cells within the TME, e.g., stromal cells (Turley et al., 2015), and suppresses cytotoxic CD8^+^ T cells through transcriptional downregulation of perforins, granzymes and a plethora of cytotoxins (Thomas and Massagué, 2005; Trapani, 2005). TGF-β also suppresses the cytotoxic activity of Th1 cells and skews T cell differentiation toward a Th2 phenotype (Turley et al., 2015). TGF-β also inhibits TCR-CD28 signaling to promote hyporesponsive memory T cells (Broderick and Bankert, 2006). CD28 is a costimulatory receptor expressed on naïve T cells that is needed for naïve T cells activation following antigen recognition. Moreover, a CD28 module is also included as a costimulatory component of recombinant engineered CARs. The impact of TGF-β on CD28 signaling in naïve and CAR- expressing T cells remains to be determined. Additionally, TGF-β is known to convert CD4^+^ T- cells into Tregs to suppress anti-tumor immunity (Nakamura et al., 2001; Trapani, 2005).

Due to its vast array of negative effects, TGF-β can impair on T cells, antibodies against it have been developed as well as T cell modifications in order to limit its negative effects on the cytotoxic activity of CAR T cells. For example, an antibody against TGF-β has been shown to positively regulate T cells (Pu et al., 2018) as well as a TGF-β inhibitor (Sow et al., 2019). A CAR that responds to TGF-β enables the genetic modification of T cells that are stimulated by this cytokine (Hou et al., 2018). Genetic ablation of the TGF-β receptor II (*TGFBR2*) in CAR T cells reduces the conversion to Tregs and to reduce T cell exhaustion. *TGFBR2*-modified CAR T cells demonstrate improved *in vivo* tumor killing using either cell line–derived or patient-derived xenografts models (Tang et al., 2020). Knocking out *TGFBRII* improved the *in vivo* function of CAR T cells in TGF-β–rich TME. Expression of a dominant negative TGF-βRII receptor (DN-TGF-βRII), inhibits downstream signaling which is required CAR T cell activity, lymphocyte proliferation, cytokine secretion, resistance to exhaustion, and increased tumor killing as shown in cancer models and a phase 1 clinical trial (Narayan et al., 2022).

Alabanza et al. (2022) created an armored BCMA CAR-T cells that expressed resistance towards. TGF-β by co-expressing a BCMA-targeting CAR with DN-TGF-βIIR armor that confers resistance towards TGF-β suppression despite its prolonged exposure. This type of armored CAR T cell design could promote durable responses in MM patients. CD28-ζ CAR T cells retain more potency in resisting TGF-β repression and improving anti-tumor efficacy (Koehler et al., 2007). It was shown that CD28DLCK-ζ CAR T cells with a hybrid IL-7Ra/IL-2Rb receptor and transgenic IL-7 exhibit superior tumor killing (Golumba-Nagy et al., 2018).

While ACT that incorporate CARs and engineered TCRs for cancer treatment are promising, efficacy and sustained response remain outstanding issues. Chimeric switch receptors (CSRs) have been developed to reverse the actions of their original signaling pathways in order to yield immune cells with the ability to overcome the immunosuppressive TME (Tay et al., 2017). Activating CSRs exploit inhibitory molecules that are expressed by cancer cells to stimulate tumor antigen-specific T cells. On the contrary, inhibitory CSRs block the effects of tumor-reactive T cells on their targets. Noh et al. developed a TGF-β/IL-7 CSR encoding the cytokine-binding portion of the TGF-β receptor extracellular domain linked to the immunostimulatory I7R signaling endodomain (tTRII-I7R). CD19 CAR-tTRII-I7R T cells displayed a reduced level of phosphorylated SMAD2 and a higher level of target-specific cell killing in the presence of recombinant human TGF-β1. In B cell lymphoma, overall and recurrence-free survival were extended in mice that received CD19 CAR-tTRII-I7R T cells compared to mice that received control (Noh et al., 2021).

## 7 TCR-expressing T cells

TCR-based ACT utilizes lymphocytes that have been genetically-engineered to target tumor-specific antigens or markers (Tsimberidou et al., 2021). The clinical use of TCR-based therapies entails a structured, multi-step process that involves patient screening and selection, generation of a transduced TCR product, lymphodepletion, and infusion of the TCR-based ACT (Zhao and Cao, 2019). Current CAR technology utilizes modularly-designed receptors introduced into immune cells. TCR-engineered immune cells use naturally occurring, or minimally modified, TCRs to develop ACTs (Figure 2). TCR-based ACT overcome the inhibitory effects of tumor-associated factors by secreting copious amounts of activated lymphocytes with known selectivity and potency. TCRs are heterodimers composed of α- and β- chains, covalently linked *via* a disulfide bridge between each chain (Huang et al., 2020). TCRs lack intrinsic signaling capacity and are activated by accessory signaling molecules (Birnbaum et al., 2014; Huang et al., 2020).

T cell activation is determined by affinity of TCR for the antigen-MHC complex. Autologous T cells have been harvested from tumors or PBMCs, expanded *ex vivo* and used as therapeutics. This approach is directed towards obtaining T cells that reflect the naturally occurring antigenic repertoire of TCRs. A recently developed approach features the genetically modified anti-tumor lymphocytes through insertion of genes that encode TCRs with defined specificity and affinity (Rosenberg and Restifo, 2015). Target antigens should be selectively expressed on tumor cells and not on normal cells (Chandran and Klebanoff, 2019).

Naïve T cells demonstrate active TGF-β signaling and TCR engagement that reduces TGF-β signaling by downregulating TGF-β type 1 receptor (TβRI) (Tu et al., 2018) (Figure 2). Moreover, SMAD7 inhibits TGF-β signaling during T cell activation (Hayashi et al., 1997; Fantini et al., 2009) (Figure 2). TCR-T cell therapies are have yielded sustained responses in patients with relapsed and/or refractory disease (Neelapu et al., 2017; Maude et al., 2018; Wang et al., 2020). Moreover, promising responses have been reported in metastatic melanoma (Rosenberg et al., 2011; Besser et al., 2013; Andersen et al., 2016; Forget et al., 2018), cervical carcinoma (Stevanović et al., 2015) and nasopharyngeal cancer (Comoli et al., 2005).

## 8 BiTE-expressing T cells directed to the TGF-β signaling pathway

Bispecific T cell engagers (BiTEs) are recombinant proteins that are comprised of two single chain variable fragments (scFv) derived from one antibody that targets a tumor-specific antigen and the other that targets CD3 on the effector T cell. Consequently, T cells are then recruited to the tumor site to target cancer cells (Goebeler and Bargou, 2020). Glioblastoma (GBM) is the most common and most lethal malignant brain tumor. Current treatment for GBM includes surgical resection, radiation and temozolomide chemotherapy, which provide only incremental benefit and are limited by systemic toxicity that damages normal brain tissue (Choi et al., 2019). Choi et al. (2019) developed a bicistronic construct to drive expression of an EGFRvIII-specific CAR and a BiTE against EGFR, an antigen frequently overexpressed in glioblastoma but also expressed in normal tissues. CART. BiTE cells secreted EGFR-specific BiTEs to redirect CAR T cells and recruit untransduced bystander T cells against wild-type EGFR. EGFRvIII-specific CAR-T cells were unable to completely treat tumors with heterogeneous EGFRvIII expression, leading to outgrowth of EGFRvIII-negative, EGFR-positive glioblastoma.

The programmed death (PD)-1 pathway is implicated in immune suppression by reducing BiTE efficacy in patients. A recent case report described a 32-year-old male patient with refractory B-precursor acute lymphoblastic leukemia (ALL) resistant to treatment with blinatumomab (Köhnke et al., 2015). BM immunohistochemistry revealed T cell infiltrates and increased PD-ligand 1 (PD-L1)-positive ALL cells as a potential immune escape mechanism. The authors recapitulated the clinical observation *in vitro* by showing that blinatumomab was not able to mediate cytotoxicity of CD19^+^ ALL cells using autologous T cells. In contrast, the addition of healthy donor T cells led to lysis of ALL cells. These results strongly support further systematic evaluation of checkpoint molecules for blinatumomab treatment failure.

TGF-β impedes the anti-PD-1/PD-L1 directed agents and promotes the emergence of drug resistance. Hence, a therapeutic strategy that prevents the effects of TGF- β on the PD-1/PD-L1 axis would be valuable to relieve or prevent drug resistance. The bispecific antibody YM101 inhibits both the PD-1/PD-L1 axis and TGF-β in murine models (Yi et al., 2021).

## 9 Conclusion and perspectives

Cancer cells, stromal fibroblasts and many other cell types that reside with the TME secrete TGF-β to promote myeloma growth and suppress anti-myeloma immunity. Consequently, the TGF-β signaling pathway impacts the anti-tumor efficacy of IMiDs, monoclonal antibodies, and T cells that have been genetically-engineered to express CARs, TCRs and BiTEs. CAR T cells offer the advantage of a single infusion with shorter treatment time, while disadvantages include cost, production, distribution, patient selection, equivocal persistence, and the generation of host antibodies against CAR antibodies. TCR T cells are generated by lentiviral transduction and circumvent the cumbersome *in vitro* production of specific immune cells. TCR T cell therapy also obviates the limitation of surface antigen expression on target cells. Barriers that limit the efficacy of CAR T cells and TCR T cells as viable, widespread options for solid tumor treatment remain significant. BiTEs combine antigen specificity with the ability to induce cytotoxicity through bystander T cells by linking an scFv that recognizes a tumor antigen and a second that recognizes CD3 on T cells. BiTE and CAR-based strategies are independent of the endogenous TCR specificity and independent of the MHC molecule on tumor surface. Cancer immunotherapy will continue to evolve and further revolutionize therapeutic strategies.

## Glossary

### The following abbreviations are used in this document:

ACT: Adoptive cell-based therapy
BiTEs: Bispecific T cell engagers
BCMA: B cell maturation antigen
BM: Bone marrow
BMSCs: bone marrow stromal cells
BMP: bone morphogenic protein
CARD11: Caspase recruitment domain-containing protein 11
CAR-T: Chimeric antigen receptor T cells
CRISPR/Cas9: Clustered Regularly Interspaced Short Palindromic Repeats And CRISPR-Associated Protein 9
CSRs: Chimeric switch receptors
CTL: Cytotoxic T lymphocyte
CTLA-4: Cytotoxic T lymphocyte antigen-4
DC: Dendritic cell
ECM: extracellular matrix
EMT: Epithelial to mesenchymal transition
Fc: Fragment crystallizable
FDA: Food and drug administration
Fox-P3: forkhead box P3
IFN: Interferon
IL: interleukin
IMiDs: immunomodulatory drugs
ITGB3: integrin β3
MDSC: Myeloid-derived suppressor cell
MHC: Major histocompatibility complex
MM: Multiple myeloma
MMP: Matrix metalloproteinase
Mo-MDSC: monocytic MDSC
mRNA: messenger ribonucleic acid
NK: Natural Killer
NKG2D: Natural Killer Group 2D
OS: Overall Survival
OTUD1: OTU domain-containing protein 1
PBMC: Peripheral blood mononuclear cell
PD-1: Programmed death 1
PD-L1: programmed death ligand 1
PFS: Progression free survival
PSMB5: Proteasome subunit beta type-5
ROS: Reactive oxygenated species
RRMM: Relapsed and/or refractory MM
SASP: Senescence-associated secretory phenotype
scFv: Single chain variable fragments
SMAD: Drosophila mothers against decapentaplegic
TAMs: Tumor-associated macrophages
TCR: T cell receptor
Teff: effector T cells
TERT: Telomerase reverse transcriptase
TGF-β: Transforming Growth Factor-beta
TGF-βR: Transforming Growth Factor-beta receptor
Th: T-helper
TNBC: triple negative breast cancer
Treg: Regulatory T cell
TME: Tumor microenvironment
VEGF: Vascular endothelial growth factor
ZEB: Zinc finger E-box-binding homeobox
